# Development of camellia oil-based beverage and its anti-atherosclerotic effects in ApoE^−/−^ mice

**DOI:** 10.3389/fnut.2026.1883934

**Published:** 2026-08-11

**Authors:** Weihua Zhang, Ning Yang, Boyu Wen, Lihui Qin, Jianhui Jiang, Yifang Wang, Mei Huang, Ruichen Wang, Lili Wu, Fuan Zhang, Guozhen Cui, Tonghua Liu

**Affiliations:** 1 School of Traditional Chinese Medicine, Beijing University of Chinese Medicine, Beijing, China; 2 School of Bioengineering, Zhuhai Campus of Zunyi Medical University, Zhuhai, Guangdong, China; 3 Guizhou Camellia Oil Engineering Technology Research Center, Tongren, Guizhou, China

**Keywords:** ApoE upper position mice, atherosclerosis, beverage, camellia oil, lipid metabolism

## Abstract

**Background:**

Atherosclerosis remains a leading cause of cardiovascular morbidity and mortality worldwide. Although camellia oil exhibits promising anti-atherosclerotic properties, its direct consumption is often limited by its strong flavor and greasy texture.

**Objective:**

This study aimed to develop a camellia oil-based beverage with a milky appearance, reduced greasiness, and enhanced palatability. In addition, the anti-atherosclerotic effects of the beverage were examined in ApoE^−/−^ mice.

**Methods:**

A camellia oil-based oil-in-water emulsion was optimized and characterized for stability, particle size, and zeta potential. Its anti-atherosclerotic effects were evaluated in high-fat diet-fed ApoE^−/−^ mice after 8 weeks of treatment using aortic Oil Red O staining, serum lipid assays, and UHPLC-Q-TOF/MS-based metabolomics.

**Results and conclusion:**

In ApoE^−/−^ mice, the beverage treatment significantly reduced aortic plaque burden, achieving an efficacy comparable to that of unprocessed camellia oil, thereby demonstrating that emulsification preserved its bioactivity. Additionally, the beverage significantly lowered serum total cholesterol, triglycerides, and low-density lipoprotein cholesterol (*p* < 0.05). Untargeted metabolomic profiling further indicated that its protective effects were associated with the modulation of metabolic pathways involved in lipid regulation. Taken together, these findings highlight the camellia oil-based beverage as a promising dietary strategy for mitigating atherosclerosis and improving lipid homeostasis.

## 1 Introduction

Atherosclerosis, a chronic inflammatory disease characterized by the accumulation of lipid-rich plaques within arterial walls, is a major cause of cardiovascular diseases and remains the primary contributor to mortality worldwide (1). This disease arises from a combination of lipid imbalances and systemic inflammation, leading to endothelial dysfunction, lipid deposition, and a persistent inflammatory response within the arterial intima (2). These processes culminate in the formation of complex atherosclerotic plaques, which may destabilize, rupture, and trigger acute cardiovascular events such as myocardial infarction and stroke. While traditional approaches to managing atherosclerosis have primarily focused on cholesterol reduction, emerging evidence suggests the need for a broader strategy that also addresses inflammation and metabolic dysfunction (3).

Dietary interventions have gained increasing attention for their potential to mitigate atherosclerosis risk factors and slow disease progression. In particular, several studies have demonstrated that diets rich in monounsaturated fatty acids (MUFAs) have cardioprotective and anti-inflammatory effects (4–6). Camellia oil (*Camellia oleifera* Abel.), often referred to as “Oriental olive oil,” is a traditional edible oil in China known for its high content of MUFAs, particularly oleic acid, along with bioactive compounds such as squalene, polyphenols, and tocopherols. Camellia oil, is a well-known food and medicine homology material, conforms to the theory of food and medicine homology, which holds that certain substances integrate pharmacological and nutritional functions into one entity (7). Studies have shown that camellia oil reduces hyperlipidemia, hepatic steatosis, and inflammation, thereby attenuating the progression of atherosclerosis in animal models (8). Notably, research using apolipoprotein E-deficient (ApoE^−/−^) mice, a widely recognized model for atherosclerosis, has demonstrated that camellia oil significantly inhibits atherosclerotic plaque formation, decreases serum cholesterol levels, and positively influences gut microbiota composition, underscoring its multifaceted role in lipid metabolism and cardiovascular protection (9).

In addition to its direct lipid-lowering effects, camellia oil has been shown to modulate gut microbiota composition, a factor increasingly recognized for its role in lipid metabolism and cardiovascular health (10–12). Studies indicate that camellia oil alters the gut microbiota in mice by increasing beneficial bacterial populations and reducing the Firmicutes/Bacteroidetes ratio, a marker associated with improved metabolic outcomes. This microbiota modulation is believed to contribute to lipid-lowering and anti-inflammatory properties, further supporting its potential role in atherosclerosis prevention (9, 11). Despite these potential benefits, direct oral consumption of camellia oil is often limited by its greasy mouthfeel, which reduces consumer acceptance. Additionally, the effective doses used in animal studies (approximately 6 mL/kg over 2 months) suggest that achieving comparable benefits in humans would require high oral intake (more than 8 mL per day), making encapsulation or direct consumption impractical (9, 11). To address these limitations, this study aimed to develop a formulated as an oil-in-water emulsion camellia oil-based beverage using monoglycerides and sucrose esters as emulsifiers, and evaluate its effects on atherosclerosis and lipid metabolism in ApoE^−/−^ mice fed a high-fat diet, as well as to explore the underlying mechanisms through metabolomic analysis.

## 2 Materials and methods

### 2.1 Materials

Camellia oil was supplied by Guizhou Yi Hang Ecological Agriculture and Animal Husbandry Technology Development Co., Ltd. (Nansi, Guizhou, China). Other ingredients used in beverage preparation, including glycerol monostearate (GMS), SE15 sucrose fatty acid ester (SE15), soybean lecithin, gum arabic powder, food-grade sodium carboxymethyl cellulose (CMC-Na), gelatin (GE), *β*-cyclodextrin (*β*-CD), sodium alginate, pectin, xanthan gum (XG), potassium sorbate, and aspartame, were sourced from Henan Wanbang Chemical Technology Co., Ltd. (Henan, China). All materials were food-grade. Kits for total cholesterol (TC), triglyceride (TG), and low-density lipoprotein cholesterol (LDL-C) were obtained from Nanjing Jiancheng Bioengineering Institute (Nanjing, China).

### 2.2 Preparation process of the camellia oil-based beverage

**Figure 1:**
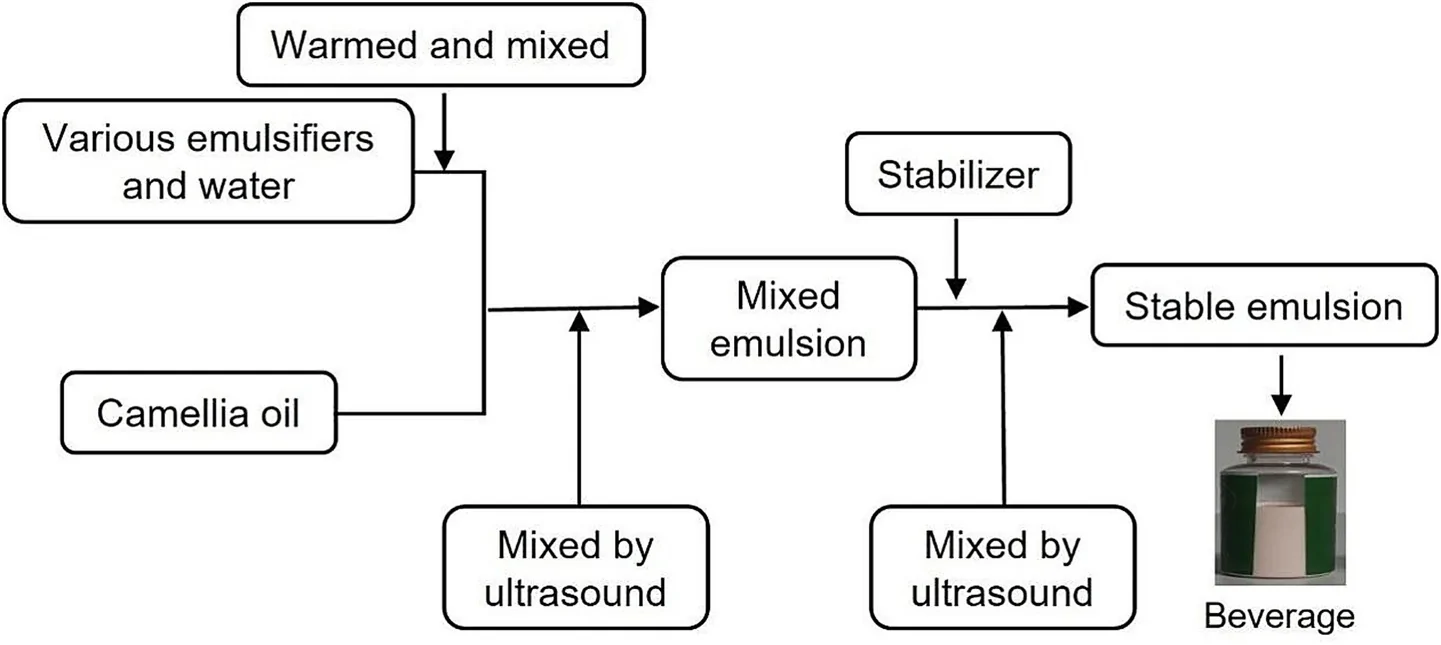
Flowchart of the preparation process for the camellia oil-based beverage.

The camellia oil-based beverage was prepared using a previously established method with minor modifications (13). The oil-to-water ratio was maintained at 3:7 (13). As shown in Figure 1, the initial process was initiated by heating 7 mL of water to 80 °C in a water bath. Upon reaching the desired temperature, emulsifiers were added and stirred until fully dissolved. Next, 3 mL of camellia oil was added to the mixture, which was then stirred continuously to facilitate emulsification, resulting in an oil-in-water (O/W) emulsion system.

### 2.3 Stability determination

The stability of the beverage was evaluated using the graduated cylinder method as previously described (14). For this evaluation, 30 mL sample was placed in a test tube at room temperature for various durations. The sample was visually monitored for phase separation into layers under the influence of gravity. When layering was observed, the ratio of the suspension thickness of the upper layer to the total height of the beverage was determined. The visual stability index (VSI) was calculated using Equation 1, where a VSI of 1 indicated no stratification.


$$\text{VSI}=1–\;\frac{\text{Height of upper suspension layer}}{\text{Total height of solution}}$$ (1)

### 2.4 Univariate analysis of excipients

For the univariate analysis of the beverage emulsifier, the performance of different emulsifiers was assessed, as the stability of the system depends on the balance between the lipophilic and hydrophilic groups of the molecules. This balance is typically quantified using the hydrophilic–lipophilic balance (HLB) of the surfactant (15). Gum arabic, a polymeric polysaccharide derived from the trunk exudate of the Acacia genus, is known for its excellent emulsifying properties. To determine the most suitable emulsifier for the beverage formulation, four emulsifiers were selected: GMS (HLB = 5), sucrose fatty acid ester (HLB = 15), lecithin (HLB = 8), and gum arabic powder. Single-factor experiments were conducted using different mass percentages of each emulsifier (0.25, 0.50, 0.75, 1, and 1.25%). After the samples were stored at room temperature for 24 h, the stability indexes were measured to evaluate the emulsifying performance of each emulsifier.

For the univariate analysis of the beverage stabilizer, the sample was mixed with an emulsifier and allowed to stand at room temperature for 72 h. Over time, phase separation and precipitation were observed, and the stability index decreased. To counteract these effects and maintain emulsion stability, stabilizers were added to increase viscosity, reduce droplet mobility, and inhibit phase separation. Six stabilizers, including xanthan gum, pectin, CMC-Na, *β*-CD, gelatin, and sodium alginate, were tested at concentrations of 0.1, 0.2, and 0.3% (mass ratio) in single-factor experiments. After the samples were stored at room temperature for 24 h, the stability indexes were determined. Based on these results, the top three stabilizers were selected for further analysis in an orthogonal experiment.

To optimize ultrasonic homogenization conditions, an ultrasonic power homogenizer (650 W, Tuohe Electromechanical Technology Co., Ltd., Shanghai, China) with a 6 mm horn was used. Sonication times (1 min, 3 min, 5 min, 7 min, and 9 min) and ultrasonic powers (40, 50, 60, and 70%) were tested to determine the optimal homogenization parameters.

### 2.5 Optimization of the emulsifier ratio for preparing the camellia oil-based beverage

After identifying the most suitable emulsifiers, two emulsifiers were combined in various ratios (from 10:0 to 0:10) to evaluate their effects. The suspension stability index was measured to determine the optimal ratio of the composite emulsifier. The HLB value is a crucial parameter in emulsion formulation, as it assists in selecting the appropriate surfactants or surfactant mixtures necessary to achieve a stable emulsified system (16). The HLB value was calculated using Equation 2 as previously described (17), where “compound A” and “compound B” refer to the respective weights of the two components. To further optimize the composition ratio of the stabilizers, an orthogonal test was performed.


$$\text{HLB value of the composite emulsifier}=\frac{\text{Compound}\;A\times \mathrm{HLB}\;A+\text{Compound}\;B\times \mathrm{HLB}\;B}{\text{Compound}\;A+\text{Compound}\;B}$$ (2)

### 2.6 Particle size and zeta potential measurement

The beverage sample (0.1 mL) was subjected to serial dilution, and the transmittance (T) of each dilution was measured using a spectrophotometer (Shanghai Spectrum Instrument Co., Ltd., Shanghai, China). The absorbance (A) was calculated using the formula A=−lg(T) as previously described (18). A dilution corresponding to 5% transmittance was selected for further analysis. The diluted sample was then introduced into the sample cell for analysis using Malvern Zetasizer Nano ZS instrument (Malvern Instruments Ltd., Malvern, United Kingdom). The zeta potential and particle diameter in nanometers (nm) were measured. Higher absolute zeta potential values (either positive or negative) indicate the presence of surface charges and the potential for electrostatic repulsion between droplets. This electrostatic repulsion contributes to the stability of the emulsion system, as higher absolute zeta potential values correlate with increased stability due to the enhanced electrostatic repulsion between particles (19).

### 2.7 Sensory evaluation

A panel of 15 trained assessors evaluated the camellia oil emulsion beverage in triplicate across four sensory attributes. The total sensory score was obtained by summing the attribute scores, with a maximum of 100 points. Assessors rinsed their mouths with warm water between samples. The scoring criteria, adapted from the Chinese national standard for plant-based beverages (GB/T 31326–2014) with modifications, are shown in Table 1.

**Table 1:** Scoring criteria for the sensory evaluation of the camellia oil emulsion.

Criteria (Max score)	Scoring standard	Score range
Flavor (30 points)	Moderate sweetness and smooth mouthfeel.	20 ~ 30
	Slightly weak or excessive sweetness; average aroma.	10 ~ 19
	Excessively strong or faint sweetness; pronounced raw off-flavor.	1 ~ 9
Mouthfeel (30 points)	Pure taste, with no greasiness or astringency.	20 ~ 30
	Acceptable taste, with slight greasiness or mild astringency.	10 ~ 19
	Poor taste, with strong greasiness or pronounced astringency.	1 ~ 9
Color (20 points)	Uniform appearance.	15 ~ 20
	Relatively uniform appearance.	10 ~ 14
	Uneven appearance.	1 ~ 9
Texture (20 points)	Uniform, smooth texture and excellent overall state. No phase separation.	15 ~ 20
	Slightly inferior state, minor but indistinct phase separation, and minimal oily suspension on the surface.	10 ~ 14
	Excessively thick or thin texture, obvious phase separation, coagulation or clumps, noticeable oily suspension, and uneven finished product.	1 ~ 9

To quantify palatability, the trained-panel scores (Table 1) were converted to a standardized 100-point scale for quantitative descriptive analysis according to previously described method with modifications (20). Smoothness was calculated by normalizing the Mouthfeel score [(Mouthfeel/30) × 100]. Greasiness and Astringency were derived inversely from the corresponding Mouthfeel and Flavor scores [100 − (Score/30) × 100]. Aroma intensity was calculated from the flavor score and capped at 80 using the equation [(Flavor/30) × 80]. This upper limit was set to account for the dilution of lipid-soluble volatile compounds by water during formulation into a camellia oil-based beverage, as well as the potential masking effects of the aqueous phase and emulsifiers in the oil-in-water (O/W) emulsion. Overall Acceptability was calculated from the Flavor and Mouthfeel scores [((Flavor + Mouthfeel)/60) × 100].

### 2.8 Animals and treatment

This study used 10 male C57BL/6 J wild-type mice and 50 male ApoE^**−/−**^ mice, all aged 6–7 weeks, with body weights of 22 ± 2 g. The animals were obtained from the Guangdong Medical Animal Experimental Center (Guangdong, China). The mice were housed at Zhuhai Campus of Zunyi Medical University under controlled conditions (temperature: 23 ± 2 °C, humidity: 60 ± 5%, and a 12-h light/dark cycle) as previously described (20). After a one-week acclimation period, 10 C57BL/6 J mice were assigned to the control group (Con, *n* = 10) and received daily oral gavage of water at a dose of 6 mL/kg. The 50 ApoE^**−/−**^ mice were randomly divided into five groups (*n* = 10 animals/group). The vehicle (Veh) group received an equal volume of water daily at a dose of 6 mL/kg. The camellia oil (Cam) group was gavaged with camellia oil at 6 mL/kg, while emulsifier mixture group (Emu) received an emulsifier mixture at 14 mL/kg. The beverage (Bev) group was administered camellia oil-based beverage at 20 mL/kg daily. A positive control group was treated with simvastatin (Aladdin Shanghai Biochemical Technology, Shanghai, China) at a dose of 5.6 mg/kg. Except for the wild-type C57BL/6 J mice, which were maintained on a standard chow diet, all the other groups were fed a high-fat diet as previously described (9). The mice were gavaged once daily for 8 weeks. Throughout the experiment, all mice had ad libitum access to food and water.

### 2.9 Pathological morphology analysis of the mouse aortic arch

The pathological morphology experiment was conducted following our previously published methods (9), using Oil Red O staining. The area of atherosclerotic lesions was quantified using Image-Pro Plus 6.0 software through blinded morphometric analysis. Lesion burden was expressed as the percentage of the lesion area relative to the aortic surface area.

### 2.10 Analysis of serum lipid biochemical indicators

After 12 h of fasting with free access to water, blood samples were collected from mice and allowed to clot at room temperature for 2 h. Samples were then centrifuged at 1,000 × *g* for 10 min at 4 °C, and the serum supernatant was collected for biochemical analysis. Serum levels of TC, TG, and LDL-C were measured using commercial detection kits in accordance with the manufacturer’s instructions.

### 2.11 Metabolomics detection and analysis

Following established methods from our previous work, a serum metabolomic study was conducted to assess biochemical changes (21, 22). Serum samples were thawed on ice to preserve their integrity. Each sample (100 μL) was mixed with 300 μL of chilled methanol (2:1, v/v), vortexed for 60 s to precipitate proteins, and centrifuged at 3,000 × *g* at 4 °C for 10 min. The supernatant was filtered through a 0.22 μm filter to remove particulates before analysis using ultrahigh-performance liquid chromatography coupled with quadrupole time-of-flight mass spectrometry (UHPLC-Q-TOF/MS, Waters Corp., Milford, United States). Chromatographic separation was performed using a Waters Acquity^™^ UHPLC system, equipped with an ACQUITY UPLC BEH C18 column (2.1 mm × 50 mm, 1.8 μm) maintained at 40 °C. The mobile phase consisted of 0.1% formic acid in water (solvent A) and acetonitrile (solvent B), and the samples were separated under the following gradient: 5–20% B (0–1 min), 20–25% B (1–5.5 min), 25–30% B (5.5–6 min), 30% B (6–8 min), 30–35% B (8–9 min), 35–65% B (9–17 min), 65–100% B (17–18 min), 100% B (18–19 min), 100–5% B (19–19.1 min), and 5% B (19.1–20 min), at a flow rate of 0.3 mL/min. The injection volume was 2 μL per sample.

Mass spectrometry was performed on a Waters SYNAPT XS system connected to the UPLC via an electrospray ionization (ESI) source operating in both positive and negative ion modes. The spectra were recorded in MSE acquisition mode, with a mass range of 50–1,200 Da. Precursor ions were fragmented using collision energies ranging from 20 to 50 eV, with a scan time of 0.5 s per spectrum. The cone voltage was set to 40 V, and the capillary voltage was set to 2.0 kV. The desolvation gas flow was maintained at 600 L/h at 350 °C, the source temperature was held at 120 °C, and the cone gas flow rate was set to 50 L/h. Lock mass calibration ensured accurate mass measurements using leucine enkephalin ions, [M + H]^+^ (m/z 556.2771) in positive mode and [M - H]^−^ (m/z 554.2615) in negative mode. Data acquisition and processing were carried out using MassLynx™ V4.1 software (Waters Corp., Milford, MA, United States). Metabolomic data analysis was performed as previously described with slight modifications (22), and Progenesis QI V2.1 software (Waters Corp., Milford, MA, United States) was used for data preprocessing. Orthogonal partial least squares discriminant analysis (OPLS-DA) and S-Plot analysis were performed using EZinfo V 3.0 software. Variables were prioritized using variable importance in projection (VIP) scores from OPLS-DA, and potential metabolites were identified based on VIP > 1, fold change (FC) > 2, and *p* < 0.05. Metabolite identification was achieved through database searches, including KEGG (23) and HMDB (24), based on accurate mass data. Pathway enrichment and metabolic pathway analyses were conducted via MetaboAnalyst 5.0 as previously described (25). According to previously reported method (26), heatmaps were generated using normalized and scaled differential metabolite abundance data, followed by hierarchical clustering to visualize group-level metabolic patterns. Spearman correlation analysis was performed to assess associations between differential metabolites and biochemical indicators, including TC, TG, LDL-C, and atherosclerotic plaque area. Spearman’s rank correlation coefficients and corresponding *p*-values were calculated, and correlations with *p* < 0.05 were considered statistically significant.

### 2.12 Statistical analysis

Statistical analysis was conducted using GraphPad Prism 9.0 software. Data are expressed as mean ± standard deviation (SD). One-way analysis of variance (ANOVA) was performed by Tukey’s multiple comparison test to assess statistical significance. *p* < 0.05 was considered statistically significant.

## 3 Results

### 3.1 Effects of the addition of raw materials and ultrasonic treatment on the stability of the beverage

**Figure 2:**
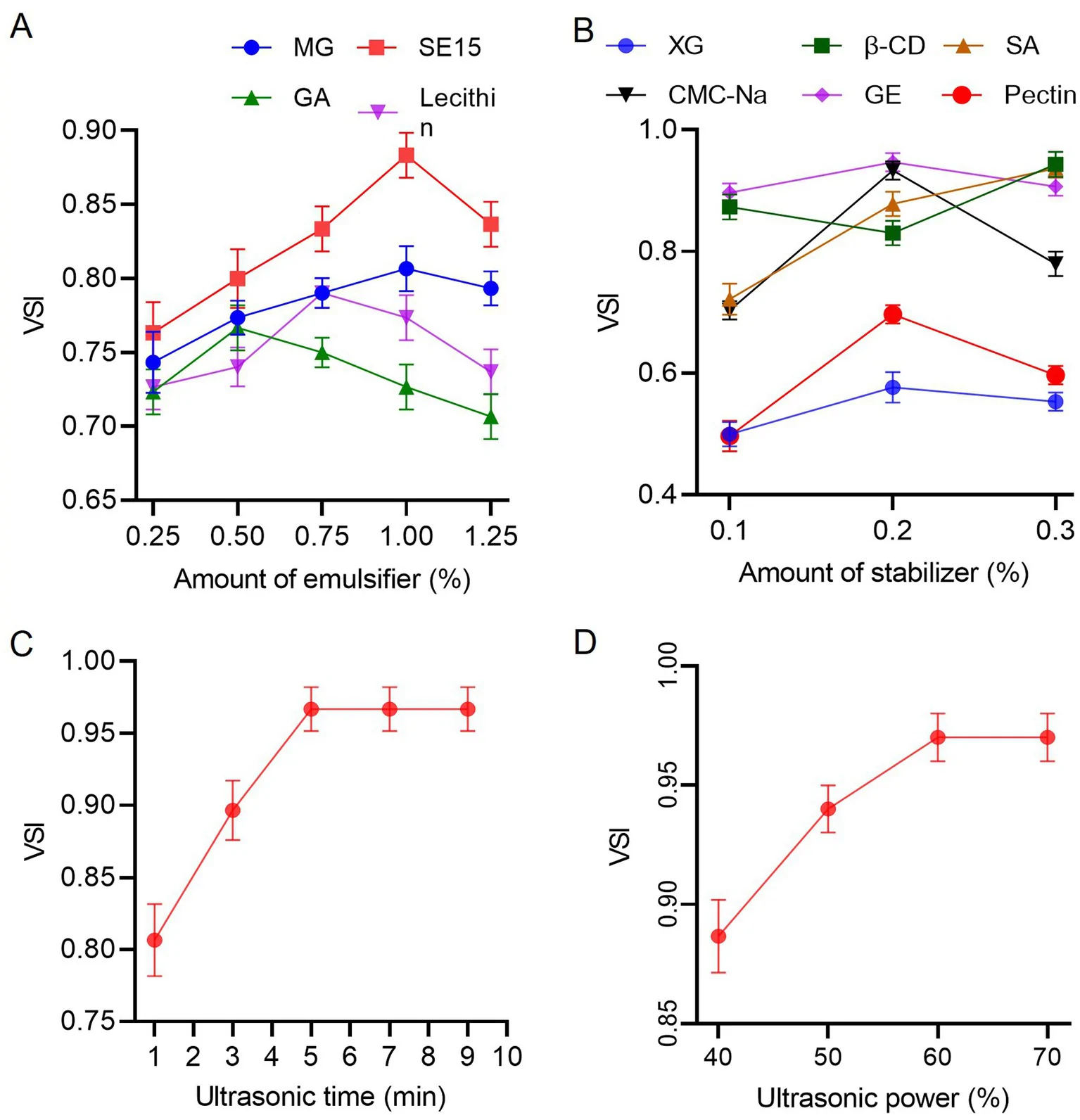
Effects of additives and ultrasonic parameters on the stability of camellia oil-based beverage. **(A)** Effects of various emulsifiers at increasing concentrations on stability factor. **(B)** Effects of various stabilizers at increasing concentrations on stability factor. **(C)** Effect of ultrasonic treatment time on VSI. **(D)** Effect of ultrasonic treatment power on VSI. MG, monoglycerides; SE15, SE15 sucrose fatty acid ester; GA, gum arabic; XG, xanthan gum; *β*-CD, *β*-cyclodextrin; SA, sodium alginate; CMC-Na, sodium carboxymethylcellulose; GE, gelatin; VSI, visual stability index.

The camellia oil-based beverage was prepared following the procedure illustrated in the flowchart (Figure 1). The effects of different emulsifiers on the stability of the camellia oil-based beverage are shown in Figure 2A. When 1% monoglyceride was added, the emulsion stability index reached a peak, indicating the best emulsification. Similarly, the addition of 1% sucrose ester also produced optimal emulsification. In contrast, the inclusion of gum arabic powder led to more oil floating in the upper layer, thickening the emulsion and causing it to cling to the container walls. Lecithin, at 0.75%, resulted in a slightly viscous emulsion, although its emulsification was less effective than that of monoglycerides or sucrose esters. Based on these findings, monoglycerides and sucrose esters were selected as emulsifiers for further experiments.

The effects of different stabilizers on the stability of the camellia oil-based beverage are shown in Figure 2B. The emulsion visual stability index (VSI) initially increased and then decreased in systems containing xanthan gum (XG), sodium carboxymethyl cellulose (CMC-Na), gelatin (GE), and pectin. In contrast, the addition of *β*-cyclodextrin (*β*-CD) resulted in an initial decrease in VSI, followed by a subsequent increase. Notably, emulsions formulated with sodium alginate (SA) exhibited a continuous increase in VSI across all tested conditions. When 0.3% sodium alginate was added, no layer separation occurred, but the viscosity was too high, making it unsuitable for beverage preparation. Thus, gelatin, *β*-CD, and CMC-Na were selected as stabilizers for further testing in a composite stabilizer formulation. Figures 2C,D illustrates the effects of ultrasonic treatment time and power on beverage stability. As both sonication time and power increased, beverage stability improved in a nearly linear fashion. The optimal stability was achieved with 60% of full ultrasonic power (650 W) for 5 min, beyond which no significant improvements were observed. Therefore, these conditions are considered optimal for preparing the beverage.

### 3.2 Effects of the emulsifier and stabilizer on the stability of the beverage

Table 2 shows the effects of various emulsifier ratios on the stability of the camellia oil-based beverage. As the proportion of GMS to SE decreased, HLB value increased, enhancing hydrophilicity and reducing surface tension. This facilitated the formation of a stable oil-in-water (O/W) emulsion. The stability index of the composite emulsifier improved accordingly. The highest emulsion stability index of 0.91 was achieved when the ratio of GMS to SE15 was 2:8. Based on these findings, this ratio was selected for further optimization in the subsequent orthogonal experiment.

**Table 2:** Effects of different proportions of composite emulsifier on the stability of camellia oil-based beverage.

GMS: SE15	HLB value	Floating volume/mL	Precipitate volume/mL	Stability index
10:0	5	5.50 ± 0.43	0.60 ± 0.23	0.80 ± 0.02
9:1	6	5.10 ± 0.45	0.60 ± 0.25	0.81 ± 0.02
8:2	7	4.90 ± 0.45	0.40 ± 0.25	0.82 ± 0.02
7:3	8	4.92 ± 0.44	0.40 ± 0.25	0.82 ± 0.02
6:4	9	4.75 ± 0.50	—	0.84 ± 0.02
5:5	10	4.30 ± 0.43	—	0.86 ± 0.01
4:6	11	3.80 ± 0.45	—	0.87 ± 0.02
3:7	12	3.45 ± 0.43	—	0.89 ± 0.01
2:8	13	2.64 ± 0.44	—	0.91 ± 0.02
1:9	14	2.85 ± 0.40	—	0.91 ± 0.01
0:10	15	3.42 ± 0.44	—	0.88 ± 0.02

“—” denotes no precipitate observed. Data are expressed as mean ± SD (*n* = 3).

### 3.3 Orthogonal test results of the stabilizers

Based on the results of the univariate experiments, an orthogonal experiment was designed to evaluate the effects of four factors on the stability of the camellia oil-based beverage: composite emulsifier (A), gelatin (B), CMC-Na (C), and *β*-CD (D). Each factor was tested at three levels, and range analysis along with analysis of variance (ANOVA) was used to evaluate the impact of these factors on the stability of the beverage. The objective of this study was to optimize the formulation of the camellia oil-based beverage. Table 3 presents the factor levels used in the experiment. According to this table, when factor A (composite emulsifier) was added at level 1 (0.5%), it was denoted as A1, with the remaining factors following the same notation.

**Table 3:** Factors and levels of the orthogonal test for the camellia oil-based beverage.

Level	Factors			
	A: composite emulsifier (%)	B: gelatin (%)	C: CMC-Na (%)	D: *β*-CD (%)
1	0.5	0.1	0.1	0.1
2	1	0.2	0.2	0.2
3	1.5	0.3	0.3	0.3

In the range analysis of the orthogonal test results for factors affecting the emulsion stability index, S1, S2, and S3 represent the sum of the indicator values for each factor at different levels, while K1, K2, and K3 represent the average values at each level. K1 was calculated by dividing the sum of level 1 values (S1) by the number of repetitions at that level. The *R*-value, which represents the range, was determined by subtracting the minimum *K* value from the maximum *K* value for each factor. The results demonstrated the following order of influence on beverage stability: C > A > D > B. Further analysis of the average values (S and K) at each level revealed that the optimal formulation was A3B2C3D3, which corresponds to 1.5% composite emulsifier, 0.2% gelatin, 0.3% CMC-Na, and 0.3% *β*-CD (see Table 4).

**Table 4:** The range analysis of the orthogonal test results for factors affecting the emulsion stability index.

Group	A: composite emulsifier(%)	B: Gelatin (%)	C: CMC-Na (%)	D: β-CD (%)	Stability index
1	1	1	1	1	0.63
2	1	2	2	2	0.81
3	1	3	3	3	0.92
4	2	1	2	3	0.95
5	2	2	3	1	0.98
6	2	3	1	2	0.75
7	3	1	3	2	0.95
8	3	2	1	3	0.92
9	3	3	2	1	0.96
S1	2.36	2.53	2.30	2.57	—
S2	2.68	2.71	2.72	2.51	—
S3	2.83	2.63	2.85	2.79	—
K1	0.7867	0.8433	0.7667	0.8567	—
K2	0.8933	0.9033	0.9067	0.8367	—
K3	0.9433	0.8767	0.9500	0.9300	—
R	0.1567	0.0600	0.1833	0.0933	—

“—” denotes not applicable.

The ANOVA results, summarized in Table 5, confirmed that CMC-Na had the most significant effect on the stability index of the camellia oil composite beverage, indicating that CMC-Na was the primary factor influencing beverage stability. Since the optimal formulation was not directly represented in the orthogonal experiment table, further analysis was conducted on two combinations: A2B2C3D1, which exhibited the highest stability index, and A3B2C3D3, identified as the optimal combination. To validate this observation, additional experiments were performed using these formulations. The reformulated beverage with A2B2C3D1 combination achieved a stability index of 0.98, which was slightly greater than the 0.97 stability index observed with A3B2C3D3 combination.

**Table 5:** Analysis of variance results for the effects of emulsifier, gelatin, CMC-Na, and *β*-CD.

Factors	SSD	Degree of freedom	Mean square	*F* ratio
A	0.0384	2	0.0192	7.11
C	0.0551	2	0.0276	10.22
D	0.0145	2	0.0073	2.70
B: (Error)	(0.0054)	(2)	0.0027	—
Total	0.1134	8	—	—

“—” denotes not applicable.

Consequently, the optimal formulation, based on the orthogonal experiment and confirmation by further testing, was determined to be 1% composite emulsifier (the ratio of GMS to SE15 = 2:8), 0.2% gelatin, 0.3% CMC-Na, and 0.1% *β*-CD. This formulation demonstrated excellent stability, without phase separation observed over a three-month period at room temperature. Analysis of the *R* values further supported the prominence of CMC-Na (*R* = 0.183) as the most influential factor, followed by the composite emulsifier (*R* = 0.157), *β*-CD (*R* = 0.093), and gelatin (*R* = 0.060). This analysis indicates that CMC-Na plays a crucial role in improving the stability of the camellia oil-based beverage. The final beverage had a milky-white appearance. Compared to camellia oil, the beverage was less greasy and had a more enjoyable mouthfeel. Collectively, the results demonstrated that formulation A2B2C3D1 provided superior stability, thereby confirming its suitability as the optimal formulation for subsequent investigations.

**Figure 3:**
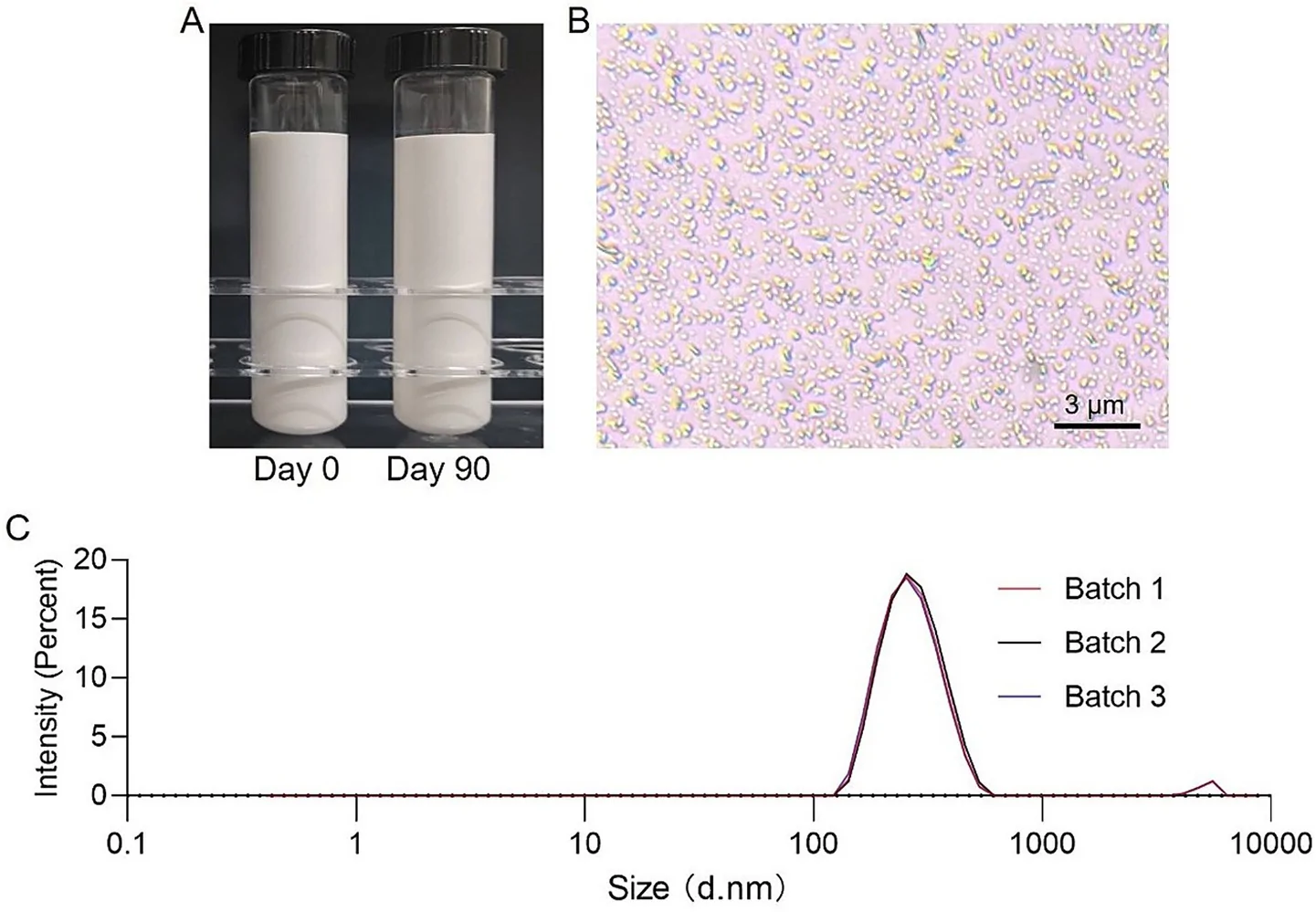
Characterization of the camellia oil-based beverage. **(A)** Representative image of the prepared beverage. **(B)** Optical micrograph showing the uniform distribution of oil droplets within the emulsion. **(C)** Particle size distribution of three independent batches, measured by dynamic light scattering (DLS). Data represent three independent experiments (*n* = 3).

The camellia oil-based beverage, once emulsified, exhibited a homogeneous, milky-white appearance (Figure 3A) and a mouthfeel comparable to that of water. Optical microscopy images revealed a uniform distribution of oil droplets in the emulsion (Figure 3B), indicating high stability. As shown in Figure 3C, three freshly prepared batches were analyzed for droplet size distribution using dynamic light scattering (DLS). The Z-average particle size was 270.0 ± 78.50 nm with a polydispersity index (PDI) of 0.183, reflecting a relatively uniform size distribution. The primary peak at 273.6 nm contributed to 98.0% of the scattering intensity, while a minor secondary peak at 5184 nm accounted for 2.0%. Zeta potential measurements revealed a value of −37.2 mV, indicating that the droplets possessed a strong negative surface charge. Notably, no visible stratification was observed during 3 months of storage at room temperature.

### 3.4 Sensory evaluation of the beverage

**Figure 4:**
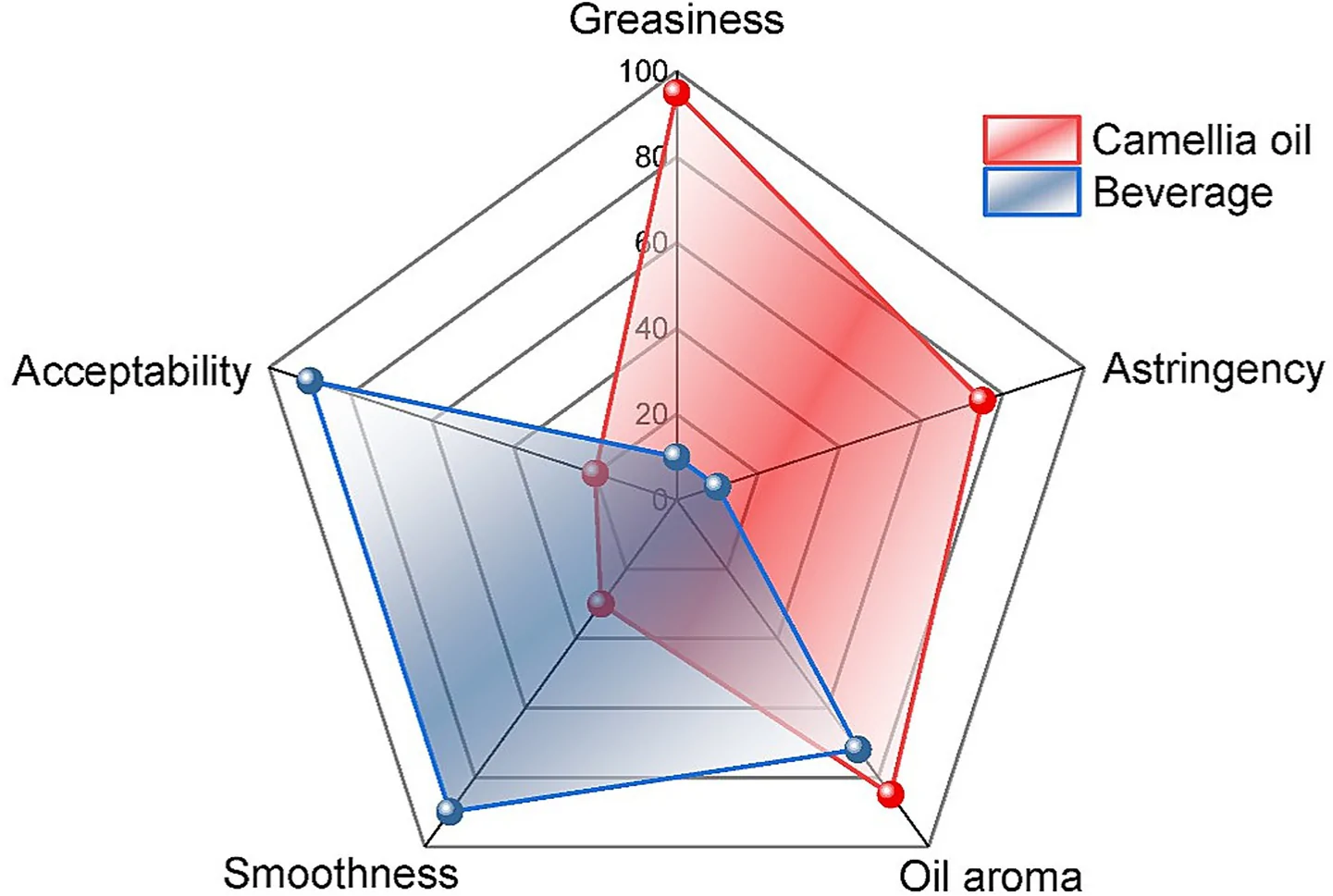
Radar chart comparing camellia oil and the beverage across five attributes.

The sensory profile of the formulated camellia oil emulsion was further quantified using empirical data collected from a trained sensory panel. As shown in Figure 4, emulsification markedly improved the sensory characteristics of camellia oil, transforming the poorly palatable raw oil into a beverage-like emulsion with high overall acceptability. Compared with pure camellia oil, the camellia oil emulsion exhibited significantly reduced greasiness and astringency. Moreover, the emulsion showed substantially enhanced smoothness and acceptability, indicating that emulsification effectively alleviated the unpleasant mouthfeel typically associated with direct oil consumption. The scores for greasiness, astringency, oil aroma, smoothness, and acceptability were 95 ± 5, 75 ± 10, 85 ± 5, 30 ± 5, and 20 ± 10 for pure camellia oil, respectively, and 9.6 ± 3.3, 10.4 ± 4.3, 71.7 ± 3.5, 90.4 ± 3.5, and 90.2 ± 3.2 for the camellia oil emulsion, respectively. These differences were statistically significant (*p* < 0.05).

### 3.5 Effects of the beverage on arterial plaques in ApoE^−/−^ mice

**Figure 5:**
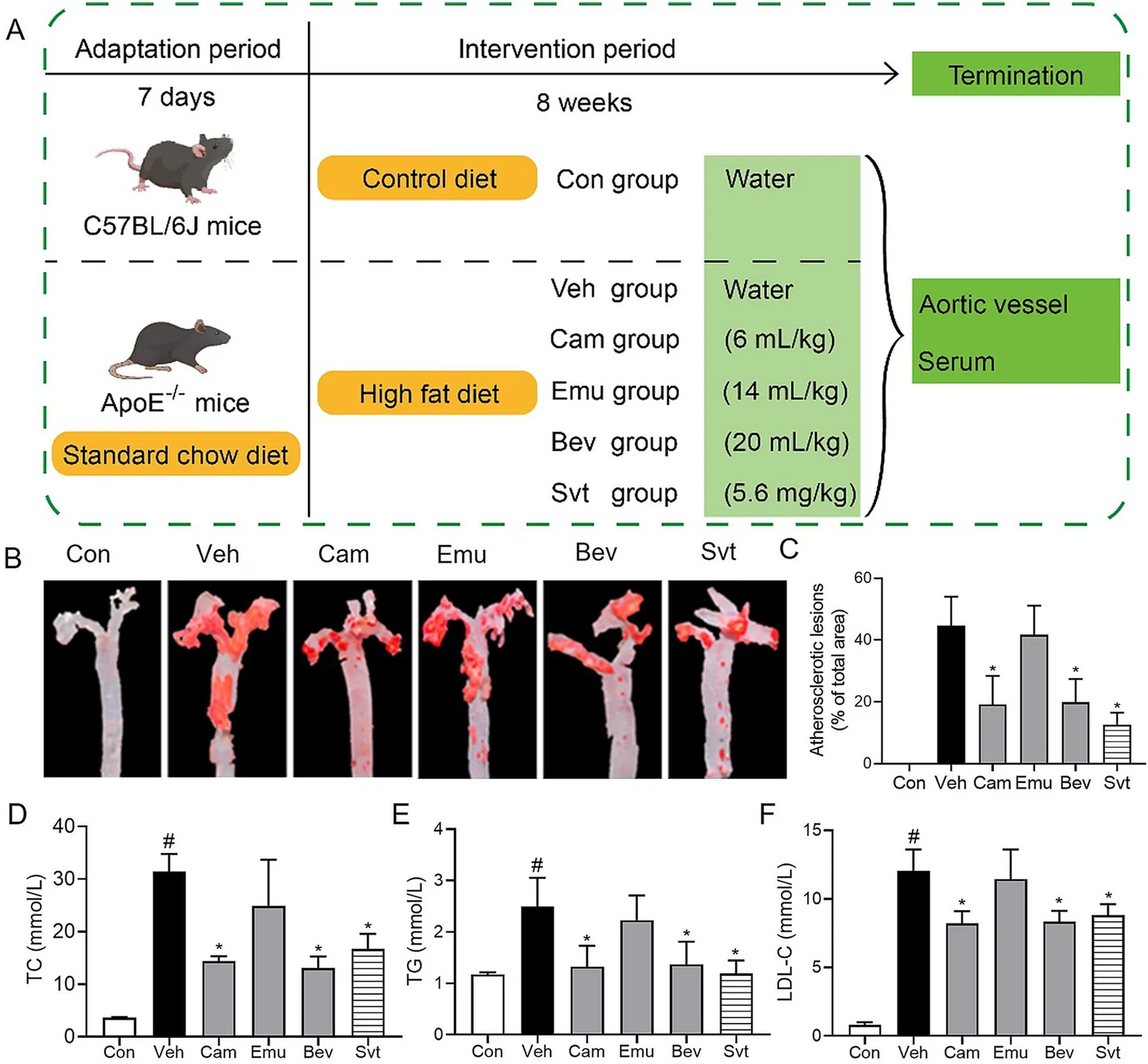
Effects of the camellia oil-based beverage on arterial plaque formation and serum biochemical indicators in ApoE^**−/−**^ mice. **(A)** Schematic representation of the experimental design and treatment regimen in ApoE^−/−^ mice administered various test samples daily. **(B)** Oil Red O staining of aortic vessels. **(C)** Quantification of aortic plaque area relative to vessel area. **(D–F)** Serum levels of TC, TG, and LDL-C, respectively. ^#^*p* < 0.05 vs. control group; ^*^p < 0.05 vs. vehicle group. Con, control; Veh, vehicle; Cam, camellia oil; Emu, emulsifier mixture; Bev, beverage; Svt, simvastatin. *n* = 10 mice per group.

The experimental design for animal treatment is shown in Figure 5A. Aortic Oil Red O staining showed the absence of detectable plaque formation in the normal control group. In contrast, ApoE^**−/−**^ mice fed a high-fat diet for 8 weeks developed severe atherosclerotic lesions throughout their aortas (Figures 5 B, C). Notably, intervention with the beverage significantly attenuated the progression of atherosclerosis, as evidenced by a quantitative analysis. It demonstrated that the ratio of the Oil Red O plaque area to the total area of intact arteries was significantly lower in the beverage group compared to the vehicle group (Figs. 5C, *p* < 0.05). Consistently, plaque area was substantially reduced in beverage-treated mice (*p* < 0.05). These findings indicate that the beverage effectively reduced atherosclerotic lesion development in ApoE^**−/−**^ mice fed a high-fat diet.

### 3.6 Effects of camellia oil-based beverage on biochemical indicators in ApoE^−/−^ mice

As shown in Figures 5D–F, the serum levels of TC, TG, and LDL-C were significantly elevated in the vehicle group compared with those in the control group (*p* < 0.05), confirming the successful establishment of the atherosclerosis model in ApoE^**−/−**^ mice fed a high-fat diet. In contrast, treatment with either camellia oil or the camellia oil-based beverage significantly reduced serum TC, TG, and LDL-C levels compared with the vehicle group (*p* < 0.05). Notably, the emulsifier control group did not significantly improve these lipid parameters compared with the vehicle group. Simvastatin, used as a positive control, also significantly decreased serum TC, TG, and LDL-C levels (*p* < 0.05).

### 3.7 Effects of camellia oil-based beverage on the metabolism of ApoE^−/−^ mice

**Figure 6:**
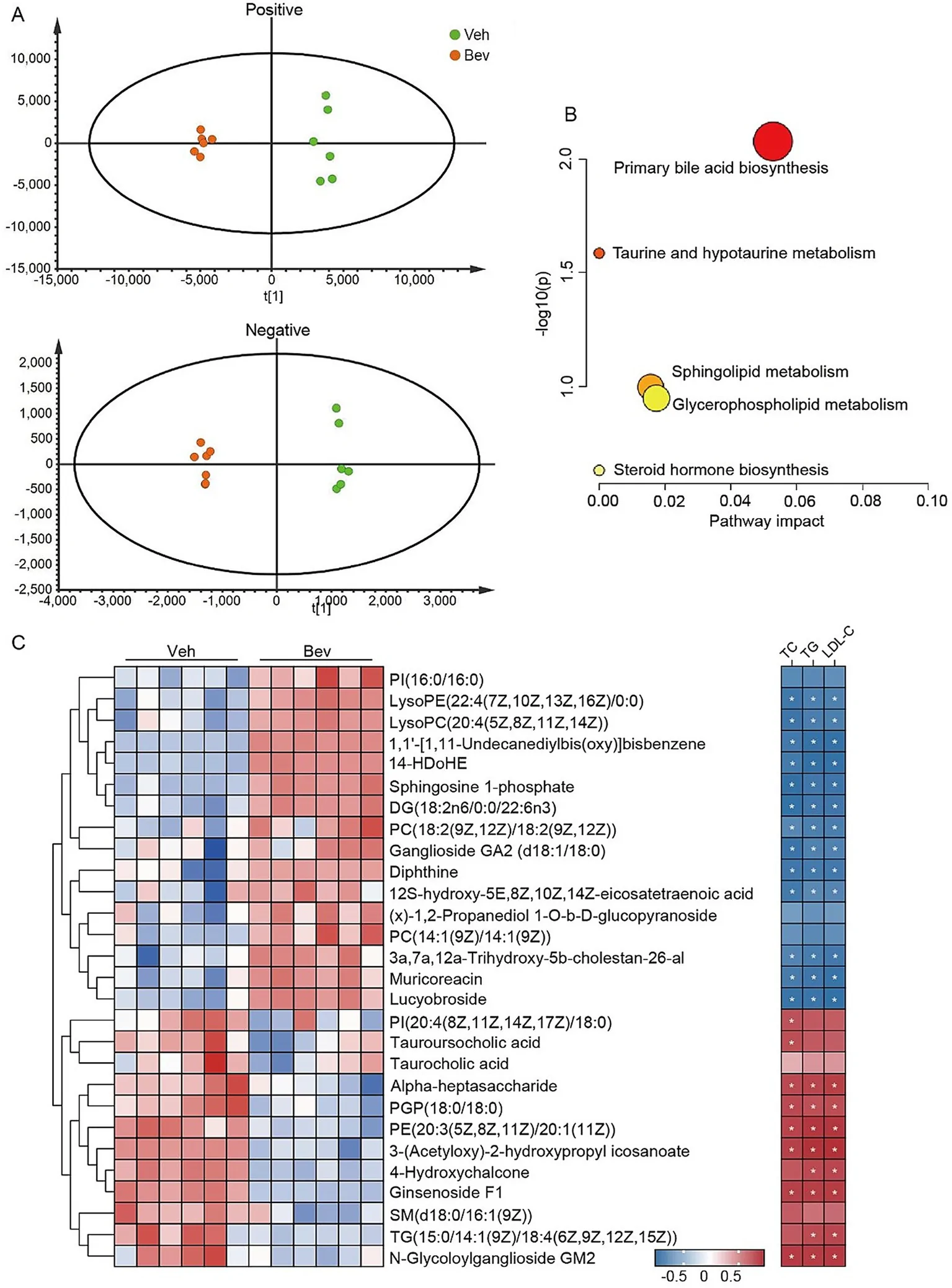
Effects of camellia oil-based beverage on the serum metabolism of ApoE^−/−^ mice. **(A)** OPLS-DA score plots and S-plots under positive and negative ion modes. The vehicle group is shown in green, and the beverage group in orange. **(B)** Pathway enrichment analysis of differential metabolites; bubble size represents pathway impact, and color intensity indicates significance level. **(C)** Heatmap of differentially abundant metabolites and their correlations with biochemical parameters (TC, TG, LDL-C). Red squares represent significant positive correlations, and blue squares represent significant negative correlations. *n* = 6 mice per group.

As shown in Figure 6A, the OPLS-DA score plot clearly revealed the separation between the two groups. The clustering of sample scatter points within each group indicated minimal within-group variability and good repeatability, while the considerable dispersion between groups indicated significant metabolic differences. The vehicle and camellia oil-based beverage groups were positioned on opposite axes, confirming substantial differences in metabolite composition in both negative and positive ion modes. Using the MetaboAnalyst database, three key metabolic pathways were identified: primary bile acid biosynthesis, glycerophospholipid metabolism, and sphingolipid metabolism (Figure 6B). Figure 6C displays a heatmap of z-score normalized metabolite intensities across the different groups. A total of 28 differentially abundant metabolites were identified based on statistical criteria (*p* < 0.05, fold change > 2, and VIP > 1). The heatmap shows distinct metabolic profiles for the vehicle and beverage treatments, as indicated by significant changes in several metabolites. Specifically, metabolites such as PI (16:0/16:0) and muricoreacin exhibited increased abundance in the treatment group, while others, including alpha-heptasaccharide and PGP (18:0/18:0), exhibited reduced abundance. Statistical significance analysis revealed the correlation between the differential abundance of these metabolites and changes in TC, TG, and LDL-C levels. For instance, metabolites such as muricoreacin and ucyobroside showed consistent patterns of increased abundance in the beverage group, accompanied by significant decreases in LDL-C and TG levels.

## 4 Discussion

To our knowledge, this is the first study to develop functional camellia oil-based beverage and assess its effects on atherosclerosis in ApoE^−/−^ mice. The optimized formulation consisted of camellia oil (oil-to-water ratio of 3:7) combined with 1% composite emulsifier (0.2% GMS, 0.8% sucrose ester SE15), 0.2% gelatin, 0.3% CMC-Na, and 0.1% *β*-CD. Importantly, emulsification did not diminish the anti-atherosclerotic effects of camellia oil in mice. The beverage significantly reduced aortic plaque formation and improved lipid metabolism by lowering the levels of TC, TG, and LDL-C. Metabolomic analysis further revealed that these benefits were associated with alterations in bile acid biosynthesis, glycerophospholipid metabolism, and sphingolipid metabolism. This study is important because it provides a scientific foundation for developing functional beverage using camellia oil as a key functional food ingredient for atherosclerosis prevention. Moreover, promoting camellia oil-based products could increase their economic value while supporting sustainable agriculture through camellia oleifera cultivation.

Our previous studies demonstrated that camellia oil benefits cardiovascular health by inhibiting arterial plaque formation. In ApoE^−/−^ mice, camellia oil reduced serum TG, TC, LDL-C, and blood glucose levels while increasing HDL-C levels and reducing arterial inflammatory markers such as TNF-*α* and IL-6, as well as liver TG and TC levels (9). The cardioprotective effects observed in the present study align with previous findings on other plant-derived oils, particularly olive oil, which is well recognized for its ability to reduce serum LDL-C levels and improve lipid metabolism (27). However, unlike traditional edible oils, the camellia oil-based beverage developed in this study offers enhanced consumer acceptability due to its emulsified form. Increased consumption of plant-based foods has been shown to reduce the risk of atherosclerosis (28). While previous studies have mainly focused on the health benefits of raw camellia oil (8, 29), our findings suggest that emulsification not only preserves its bioactivity but also improves its palatability, making it a more practical option for functional food applications. As far as we know, this is the first study to develop functional oil-based beverage with anti-atherosclerotic potential. The camellia oil-based beverage showed comparable efficacy in ApoE^−/−^ mice while improving emulsion stability, reducing greasiness and enhancing palatability, supporting its promise as a consumer-friendly functional lipid intervention. Natural Nrf2 activators, including polyphenols, sulforaphane, curcumin, resveratrol and tea polyphenols, have been linked to cardiovascular protection through regulation of antioxidant defense, inflammation and lipid metabolism (30). Camellia oil contains polyphenols, tocopherols and other antioxidants that may contribute to vascular protection (31). However, Nrf2 signalling and downstream antioxidant enzymes were not directly assessed in this study. Thus, Nrf2-mediated regulation remains a plausible mechanism for future investigation rather than direct evidence from the present data. Future work should determine whether the beverage modulates Nrf2, HO-1, NQO1, and related oxidative-stress pathways in vascular, hepatic, and intestinal tissues.

In the preparation of the camellia oil-based beverage, the oil was incorporated as an oil-in-water emulsion. The stability of such emulsions is influenced by several factors, including the type of emulsifier, the concentration of stabilizers, and the processing methods applied. Emulsifiers and stabilizers play crucial roles in preventing phase separation and ensuring product consistency (32). Advanced techniques such as high-pressure homogenization and encapsulation are being explored to improve the bioavailability and stability of oil-based compounds, ensuring that the final product meets consumer expectations for both nutritional and sensory appeal (33). The stability of the emulsion depends on the strength of the interfacial film formed by surfactant molecules at the oil–water interface (34), with more coherent and densely packed films conferring enhanced stability. For instance, the presence of mixed surface-frozen films formed by surfactants and coexisting organic molecules can further improve the emulsion’s kinetic stability (35). In this study, the stability of the beverage increased with the addition of stabilizers, reaching an optimal level before declining. This improved stability is attributed to enhanced steric hindrance and repulsion, which prevents phase separation. However, excessive stabilizer concentrations led to decreased solubility, coagulation, and stratification. High-pressure homogenization effectively reduced the particle size, improved the texture, and prevented fat separation and sedimentation. Furthermore, ultrasonic treatment reduced particle size, enhancing uniformity and stability (36). Our findings emphasize the effectiveness of adding stabilizers post-emulsification to enhance the stability of the camellia oil emulsion. Oil–water emulsions can also inhibit lipid oxidation, with surfactants such as proteins, polysaccharides, and phospholipids playing a key role in controlling the oxidation of polyunsaturated fatty acids, thus preserving emulsion stability (37). Emulsifying camellia oil may therefore not only improve its solubility and bioavailability but also protect it from oxidation, which is triggered by external factors. These characteristics are important for functional food development because consumer compliance is influenced not only by biological efficacy but also by taste, mouthfeel, convenience, and product stability. Therefore, the present formulation provides a useful foundation for developing camellia oil into a more acceptable daily dietary intervention rather than relying on direct oil intake, which is often limited by its greasy texture and poor sensory appeal.

The findings of this study also provide several practical implications for future functional food development. First, formulation optimization should focus on maintaining an appropriate oil-to-water ratio, selecting compatible emulsifiers and stabilizers, and controlling processing parameters to balance emulsion stability, sensory quality, and bioactivity. In the present study, the use of an oil-in-water emulsion system improved dispersion of camellia oil and reduced greasiness, indicating that emulsion-based delivery may be an effective strategy for incorporating lipid-soluble bioactive ingredients into beverage products. Second, future product development should further optimize flavor masking, sweetness, viscosity, droplet size distribution, and storage stability to enhance consumer acceptance and commercial feasibility. Third, although the present animal study used a defined gavage dose to evaluate biological efficacy, this dose cannot be directly translated into a recommended human intake. Future studies should therefore include dose–response experiments and human equivalent dose calculations to identify a safe and effective intake range for dietary use.

From biological perspective, the camellia oil-based beverage significantly reduced atherosclerotic plaque formation in ApoE^−/−^ mice, demonstrating similar efficacy to that of raw camellia oil. This finding suggests that emulsification did not compromise its bioactivity. Moreover, metabolomic analysis revealed that beverage treatment modulated key metabolic pathways, including bile acid biosynthesis, glycerophospholipid metabolism, and sphingolipid metabolism. This observed trend is similar to that observed in ApoE upper position mice treated with camellia diacylglycerol oil (38). Research has shown that bile acids play a critical role in lipid digestion and act as signaling molecules that regulate glucose and lipid metabolism (39, 40). Additionally, bile acids are recognized as key mediators of cardiovascular health, influencing vascular tone, cholesterol homeostasis, and inflammatory responses (41).

Glycerophospholipids, the most abundant phospholipids in the body, play essential roles in maintaining cell membrane integrity and regulating cellular signaling (42). Their breakdown products, such as lysophosphatidylcholine, are known to influence inflammation and metabolic disorders (43). Our findings suggest that camellia oil modulates these pathways by reducing the levels of pro-inflammatory lipid metabolites, thereby supporting lipid homeostasis and reducing atherosclerotic lesion burden in mice. The ability of emulsified camellia oil to positively affect lipid metabolism indicates its potential as a dietary component for atherosclerosis prevention. In addition to its applications in the food industry, this research provides valuable mechanistic insights into how camellia oil affects lipid metabolism. Overall, this study contributes to the broader field of functional food innovation and paves the way for future research on plant-derived bioactive compounds for disease prevention.

Despite these promising results, this study has certain limitations. First, the relevance of these findings to human physiology remains uncertain, and clinical trials are needed to validate the efficacy, optimal dosage, and bioavailability of the camellia oil-based beverage for preventing atherosclerosis in humans. Second, edible oils are susceptible to oxidation over time, leading to decreased nutritional quality and the formation of harmful compounds (44). Factors such as fatty acid composition, heat, and light exposure influence oil stability (45). Given previous evidence that emulsification protects menhaden oil from oxidation (46), emulsification and encapsulation may offer similar protective effects for camellia oil, but further studies are needed to confirm this. Lastly, although metabolomics implicated bile acid, glycerophospholipid, and sphingolipid pathways, the causal mechanisms remain to be clarified. Targeted validation should focus on mediators suggested by our findings, including conjugated bile acids, lysophosphatidylcholines, sphingolipid metabolites, and related molecular regulators such as FXR, TGR5, CYP7A1, and LPCAT. Examination of these candidates through targeted metabolomics, expression analyses and functional perturbation experiments will be essential for linking metabolite signatures to underlying mechanisms in future studies.

## 5 Conclusion

This study demonstrated that the camellia oil-based beverage effectively reduces atherosclerosis and improves lipid metabolism in ApoE^−/−^ mice. The optimized emulsion formulation preserved the functional properties of camellia oil while enhancing its palatability and consumer appeal. Metabolomic profiling indicated modulation of lipid-related pathways, supporting the potential of this formulation as a cardioprotective functional food candidate. Further investigation, including human intervention studies, is warranted to evaluate its translational relevance in dietary strategies for atherosclerosis prevention.

## Data Availability

The original contributions presented in the study are included in the article/supplementary material, further inquiries can be directed to the corresponding authors.
